# Novel Insights into Postoperative Surveillance in Resected Pancreatic Cystic Neoplasms—A Review

**DOI:** 10.3390/diagnostics14101056

**Published:** 2024-05-19

**Authors:** Daniel Vasile Balaban, Laura-Ioana Coman, Marina Balaban, Raluca Simona Costache, Mariana Jinga

**Affiliations:** 1 Internal Medicine and Gastroenterology Department, Carol Davila University of Medicine and Pharmacy, 020021 Bucharest, Romania; laura.coman21@yahoo.com (L.-I.C.); raluca.costache@umfcd.ro (R.S.C.); mariana.jinga@umfcd.ro (M.J.); 2 Gastroenterology Department, Central Military Emergency University Hospital, 010825 Bucharest, Romania; 3 Doctoral School, Carol Davila University of Medicine and Pharmacy, 020021 Bucharest, Romania; marina.ciochina@drd.umfcd.ro

**Keywords:** pancreatic cyst, neoplasm, resection, surveillance, recurrence

## Abstract

Pancreatic cystic lesions (PCL) are frequently encountered in clinical practice and some are referred to surgery due to their neoplastic risk or malignant transformation. The management of PCL involves complex decision-making, with postoperative surveillance being a key component for long-term outcomes, due to the potential for recurrence and postoperative morbidity. Unfortunately, the follow-up of resected patients is far from being optimal and there is a lack of consensus on recommendations with regard to timing and methods of surveillance. Here, we summarize the current knowledge on the postoperative surveillance of neoplastic pancreatic cysts, focusing on the mechanisms and risk factors for recurrence, the recurrence rates according to the initial indication for surgery, the final result of the surgical specimen and neoplastic risk in the remaining pancreas, as well as the postsurgical morbidity comprising pancreatic exocrine insufficiency, metabolic dysfunction and diabetes after resection, according to the type of surgery performed. We analyze postsurgical recurrence rates and morbidity profiles, as influenced by different surgical techniques, to better delineate at-risk patients, and highlight the need for tailored surveillance strategies adapted to preoperative and operative factors with an impact on outcomes.

## 1. Introduction

Pancreatic cystic neoplasms comprise a wide spectrum of lesions with a highly variable risk of progression to malignancy. Despite the publication of several guidelines to guide clinical practice [1,2,3,4,5,6], and substantial improvements in imaging techniques, diagnosis and decision-making regarding a pancreatic cystic lesion (PCL) can be challenging and sometimes erroneous [7,8].

After evaluating a PCL, the clinician has several options, each with associated potential pitfalls. The patient can either be monitored at different time intervals according to the adopted guideline [9], which carries the risk of missing early cancer and the burden of frequent follow-up tests, or they can be referred for surgery, which poses the risk of the patient undergoing an unnecessary major surgical intervention for what might be a benign lesion due to an inconclusive or false-positive preoperative assessment. Also, in patients unfit for surgery, there are recommendations for stopping the surveillance of PCLs [2,5,6,10]; however, this is subject to change given the development of minimally invasive therapies such as endoscopic ultrasound (EUS)-guided ablation techniques, which show promising results in published series [11,12]. 

A surgical decision for a PCL can sometimes be challenging, as malignancy is not always evident on preoperative tests and pancreatic surgery is associated with major mortality and morbidity, which significantly impact patients’ quality of life [13]. The European-based guidelines [2] propose absolute and relative indications for surgery, with the latter additionally taking patients’ comorbidities into account. In the process of decision-making, clinicians must also consider the performance status of patients and some negative prognostic factors for pancreatic surgery, such as advanced age, overweight or diabetes mellitus (DM) [14]. On the other hand, as an alternative to prolonged surveillance, in young, fit patients with intraductal papillary mucinous neoplasm (IPMN) over 3 cm, surgery can be considered [1]. Patient selection is of paramount importance, as mortality due to pancreatic surgery is not negligible, and morbidity is significant and frequently under-evaluated. Decision-making according to the current guidelines has proven to result in a significant rate of benign resections, with a tendency of overtreatment, reflected in excessive surgery in presumptive malignant cysts which were not found to be cancerous after surgery [14,15,16,17,18,19,20]. However, other surgical series have highlighted the risk of undertreatment, translated into the potential of missing a diagnosis of cancer [21,22]. 

Moreover, in cases where the surgical specimen is confirmed to be a premalignant or malignant PCL, there is the issue of surveillance after surgery. The need for surveillance has further increased as a side benefit of improved surgical outcomes, including increased survival after pancreatectomy. However, tailored surveillance based on recurrence risk is required in resected PCLs, because indefinite monitoring carries a high burden for healthcare systems, including significant costs [23].

**Figure 1:**
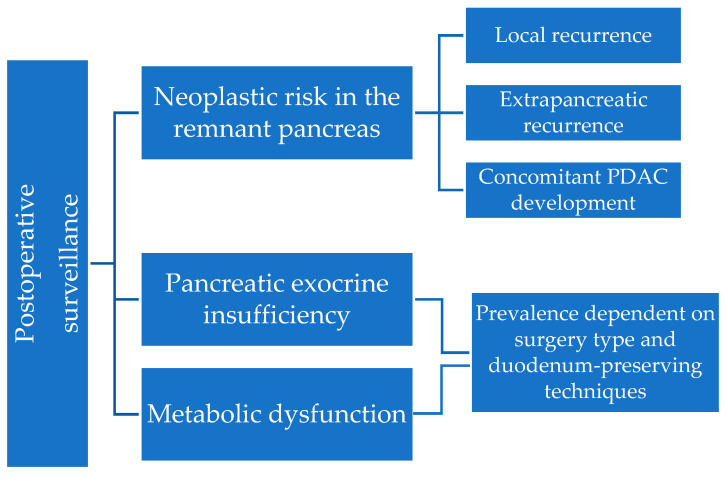
Approach to surveillance after pancreatic cyst resection.

There is abundant literature on optimizing the surveillance of PCLs, focusing on reducing the burden of trivial cysts and the accurate detection of malignancy for at-risk ones [24,25,26], but less on the follow-up of resected cysts. In this review, we aim to summarize the current recommendations concerning surveillance methods and intervals in resected PCLs, according to the initial indication for surgery, the postoperative histopathological report and the neoplastic risk in the remaining pancreas. Also, we discuss the exocrine and endocrine consequences after pancreatic resection, particularly pancreatic exocrine insufficiency, but also metabolic dysfunction, including DM and hepatosteatosis, as shown in Figure 1. We will not cover operative mortality and short-term morbidity, representing early complications of pancreatic surgery.

## 2. Search Strategy

For the purpose of this review, we performed a Pubmed search in September 2023 for publications referring to the surveillance of resected pancreatic cysts, using the medical subject heading (MeSH) term “Pancreatic Cyst/surgery”[Mesh] (ID: D010181), in association with the following keywords: “surveillance”, “follow-up”, “recurrence”, from 2003 onwards. Relevant papers were selected after screening the title and abstract content. Also, references of pertinent studies and their citing articles were further assessed to identify additional relevant papers that were missed by the initial search. Papers that came to the authors’ attention by means of personal research or scientific platforms were also considered.

## 3. Surveillance of the Remnant Pancreas

Surveillance of the remnant pancreas is frequently not adequately carried out after surgery for a PCL. Firstly, there is heterogeneity amongst the published studies regarding the definition of a remnant pancreatic lesion. Moreover, surveillance intervals, methods and indications depend on several factors, such as the indication for surgery (neoplasia; pre-neoplastic lesions), the final histopathological result of the resected specimen, the magnitude of neoplastic risk in the remaining parenchyma and the recurrence risk at anastomosis. 

### 3.1. Mechanisms and Risk Factors for Recurrence 

Recurrence is well described in resected intraductal papillary mucinous neoplasms (IPMNs), occurring, on average, in 11–20%, with higher rates in malignant lesions [27]. In non-invasive IPMNs, the median recurrence rate, according to a recent systematic review, is 8.8% (0–27.6%) [28]. 

Recurrence may occur within the remaining pancreatic tissue, known as intrapancreatic recurrence, or outside the pancreas, termed extrapancreatic recurrence. This latter form has been reported in instances of IPMN associated with invasive carcinoma [29].

**Figure 2:**
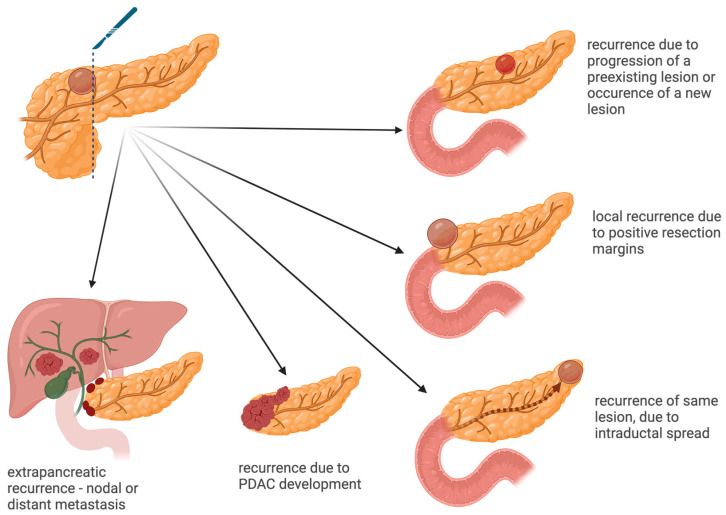
Mechanisms of recurrence in resected pancreatic cystic neoplasms (Created with BioRender.com, accessed on 8 April 2024).

After surgical resection, the “unstable” ductal epithelium can give rise to further cystic lesions, or even a ductal adenocarcinoma [30]. Several mechanisms have been theorized to account for IPMN recurrence in the remnant pancreas [31,32], as presented in Figure 2:

- Recurrence of the initial lesion—either through positive margins after surgery, with residual microscopic disease which progresses over time, or through the intraductal spread of neoplastic cells, which leads to a new lesion in the remaining parenchyma, distant from the resection site, but with a similar genetic background.
- Progression of multifocal disease—either through the progression of residual lesions, which were accurately detected preoperatively but did not show indication for resection, or through the occurrence of a genetically non-related neoplastic lesion, independently of the index cyst.

The progression of preexisting lesions and the occurrence of new cysts are the most common types of recurrences in resected IPMNs [33]. Risk factors for recurrence, as highlighted in several surgical series, are as follows [34,35,36,37,38]: family history of pancreatic cancer, preoperative symptoms, body and tail of the pancreas as the dominant location of IPMN, dilated main pancreatic duct (MPD) ≥ 10 mm, multifocal lesions, high-grade dysplasia (HGD) or invasive carcinoma on histology. Recurrence at the site of anastomosis is of particular interest, and for advanced lesions (HGD or invasive carcinoma), close follow-up is warranted [39]. 

Besides local recurrence, there is also the risk of extrapancreatic recurrence, which is defined by nodal or metastatic disease. Risk factors for systemic recurrence are represented by invasive lesions, mixed-type IPMNs, poor differentiation, nodal disease, elevated serum CA 19-9 and intraoperative transfusion [40,41].

### 3.2. Initial Indication for Surgery and the Results of the Resected Specimen

Surgical indications in PCL are limited to evidence of malignancy or risk of malignancy on preoperative tests, or, in particular cases, the impossibility of excluding malignancy after an equivocal cyst workup, following the decision of a multidisciplinary board. In these latter cases, a molecular analysis of cyst fluid can support the surgical decision [42,43]. According to European guidelines, surgery for IPMN is firmly recommended if there is evidence of malignancy or HGD, the presence of a solid mass, tumor-related jaundice, enhanced mural nodule ≥ 5 mm or dilatation of MPD ≥ 10 mm. A moderate dilatation of MPD (5–9.9 mm), cyst size ≥ 40 mm or growth rate ≥ 5 mm/year, increased CA 19-9 > 37 U/mL (without jaundice), enhanced mural nodule < 5 mm, flare in acute pancreatitis related to IPMN and new-onset diabetes (NOD) are regarded as relative indications for surgery, and should be evaluated in association with comorbidities in elderly, frail patients [2]. In the recent Kyoto guidelines [6], the absolute indications from the EU guideline are referred to as “high-risk stigmata” (HRS) for HDG/invasive cancer, as initially termed in the 2012 Fukuoka consensus [4], and the relative indications correspond to the “worrisome features” (WFs) from the 2017 revised Fukuoka guideline, with the addition of cyst growth rate and new-onset/exacerbation of DM. Nomograms can be used for risk stratification and surgical decision-making by assessing all clinical and imaging WF, as the presence of multiple WFs increases the probability of HGD/invasive cancer up to 100% in patients with ≥4 factors [44]. In the ACG guidelines, multidisciplinary referral for consideration for surgery is recommended in cases of positive cytology for HGD/cancer, cyst-related jaundice or acute pancreatitis, significantly elevated CA 19-9 or concerning features on EUS, and for solid pseudopapillary neoplasms [5]. As for PDAC, biological and conditional factors should be taken into account when deciding on PCL surgery, and in patients with a long life expectancy but who are unfit or have contraindication for surgery, ablation therapy might be considered.

The surgical strategy for a PCL is decided according to the location of the cyst and the preoperative assessment of cyst type. The 2023 Kyoto guidelines for the management of IPMN provide indications for specific surgical techniques according to IPMN subtype and evidence or suspicion of malignancy, as shown in Table 1 [6].

The recurrence and survival rates are strongly dependent on the final histological specimen and resection margins [45]. In case of evidence of malignancy at the resection margins (HGD or invasive carcinoma), an additional resection is required to achieve negative or at least LGD margin. Higher recurrence and lower survival rates have been reported in invasive IPMN compared to HGD and LGD-IPMN [46]. Notably, even non-invasive IPMNs are at risk of progression—in the study by Amini et al. [37], 44% of patients who developed invasive carcinoma had only LGD on the index resection specimen. Regarding histological subtype, oncocytic IPMNs seem to be correlated with a good outcome [45]. Another subgroup with favorable prognosis is represented by non-invasive side branch-IPMN, with infrequent recurrences [45]. In addition to histological subtype, another important feature influencing recurrence is represented by genetic alterations such as Kras and GNAS mutations [47]. Also, the pattern of mucin (MUC) expression seen with different IPMN subtypes might be correlated with recurrence risk [48].

While preoperative imaging is used to plan the surgery and intraoperative frozen sections are routinely performed to assess the resection margins [49], the lesion’s extent can be more accurately determined by using pancreatoscopy, a minimally invasive technique which can potentially delineate “skip” lesions in the pancreatic ducts [50]. A discontinuous pattern of lesions can be missed by standard preoperative tests and has been reported in up to 10% of IPMNs in surgical series [49]. In a systematic review and meta-analysis from the European Cholangioscopy study group in 2023, pancreatoscopy proved to have a high diagnostic accuracy, leading to a change in clinical management in 13–62% of patients, but at the cost of a significant rate of adverse events (12%, mostly pancreatitis of mild severity) [51].

Patients with surgically resected benign cysts, such as pseudocysts, serous cystadenoma or mucinous cystic neoplasm (MCN) without associated invasive carcinoma, do not require postoperative follow-up. In patients who underwent resection for a solid pseudopapillary neoplasm, the recommendation is yearly surveillance for at least 5 years [5]. 

### 3.3. Surveillance Intervals and Methods 

The clinical impact of surveillance of the remnant pancreas after resection is not very well represented in the literature. In a large series from Japan, repeat pancreatectomy for secondary lesions after initial resection was carried out in 1.4% of cases [35]. Other studies have reported higher reoperation rates, ranging from 8 to 11% [37,38]. The majority of recurrences occur in the first 3 years after surgery, when intensive surveillance is warranted, but they can also develop at a later time, supporting the need for long-term follow-up of these patients [32,36]. 

Regarding the frequency of surveillance, there is wide variation among studies reporting on this criteria—for IPMN, some have proposed intensive monitoring, at 3–6-month intervals for the first two years, followed by 6–12-month intervals; others have opted for looser follow-up intervals [2,28,52,53]. Follow-up in resected IPMN is lifelong or stopped at the point where the patient is unfit for or unwilling to undergo surgery, although some patients might be candidates for EUS-guided ablation therapies. In resected MCNs, surveillance is required if invasive carcinoma is present [53]. The presence of HGD and the multifocality of cysts defines a high-risk group which might benefit from more intensive monitoring [37]. In case of invasive IPMN on the resection specimen, surveillance should be carried out similarly to monitoring after resected pancreatic ductal adenocarcinoma (PDAC) [32]. 

There is no consensus on the surveillance modalities either—while biomarkers play a limited role, the main techniques are represented by cross-sectional imaging (CT/MRI) and EUS, with an additional contrast enhancement [28,32]. Imaging should focus on detecting high-risk stigmata, as defined for IPMNs, consisting of the presence of a solid mass or main pancreatic duct dilation, although the latter can also be related to the stricture of pancreato-jejunal anastomosis [54]. With regard to biomarkers, while CA 19-9 has been widely studied due to its prognostic value after pancreatic cancer resection and in monitoring PCLs [55,56], its role in the follow-up of patients with resected PCLs is less studied and it seems to make less of a contribution [34,40,57,58]. In the surgical series which report on the use of biomarkers in postoperative surveillance, tumor markers were assessed at 3–6-month intervals. In the study of Miyasaka et al. [58], among the thirteen patients with the metachronous development of high-risk lesions in the remnant pancreas, there were five patients with elevated tumor markers (three of them had both increased CEA and CA 19-9, and two had increased CEA or CA 19-9 only). Further research should explore the dynamics of the currently available serum biomarkers and the development of novel ones that can predict recurrence with better accuracy. With the promising data from PDAC diagnosis by means of liquid biopsy, circulating cell-free DNA and microRNAs might also play a role in the surveillance of resected pancreatic cystic neoplasms [59]. 

The field of multi-omics has made significant progress over the last twenty years, primarily attributed to technological advancements that have facilitated the efficient and high-capacity examination of biological molecules (microRNAs, genetic and epigenetic mutations, protein markers, markers of metabolomic alterations, etc.). Certain omics fields are exhibiting promising capabilities in the quest for a new biomarker for PDAC. However, the current information available for PCLs is considerably restricted [60]. A recent study reported the development of an algorithm based on biomarker risk scores to improve risk stratification in patients undergoing surgery and/or surveillance for a PCL. Combinations of cyst fluid biomarkers with reported evidence of high specificity (>85%) for distinguishing PCLs were used to reinforce confidence in a preoperative diagnosis, which is critical for patients undergoing surveillance. For non-mucinous SCNs, vascular endothelial growth factor (VEGF) > 5000 pg/mL, glucose > 50 mg/dL, CEA < 10 ng/mL and amylase < 250 U/L were used, while MCNs were classified by glucose < 50 mg/dL, CEA > 192 ng/mL, cytology (mucinous) and the presence of mutations (KRAS/GNA). Within the surgical cohort, the algorithm demonstrated a superior performance overall compared to the preoperative clinical diagnosis in accurately predicting the final pathological diagnosis (91% vs. 73%; *p* < 0.000001), exhibiting a higher rate of the correct classification of non-mucinous SCNs and MCNs compared to clinical diagnosis (96% vs. 30%; *p* < 0.000008 and 92% vs. 69%; *p* = 0.04, respectively). Moving on to the surveillance cohort, the algorithm displayed the capacity to forecast a preoperative diagnosis with a significant level of certainty, grounded upon a substantial biomarker score and/or alignment with imaging data from at least one follow-up visit, supporting the clinical utility of the use of biomarker for PCL surveillance [61]. However, further validation studies for the use of biomarkers in patient populations with PCLs are needed, focusing on predicting recurrence and outcomes in the surveillance of resected cysts. 

## 4. Pancreatic Exocrine Insufficiency after Pancreatic Surgery for PCL

Pancreatic exocrine insufficiency (PEI) is a common consequence of pancreatic surgery performed for both benign and malignant pathology. A systematic review and meta-analysis by Beger et al. [62], including 2729 patients, found that after a mean follow-up of 32 months after pancreatic surgery, 44.9% of patients developed new-onset PEI. Several factors have been analyzed in relation to PEI occurrence after pancreatic surgery, which we will further detail. Concerning both endocrine and exocrine pancreatic dysfunction, a limitation of the currently available data is that most of the published surgical series do not refer to PCLs only, but include a wide spectrum of benign and malignant pancreatic lesions. 

### 4.1. Type of Surgery

**Figure 3:**
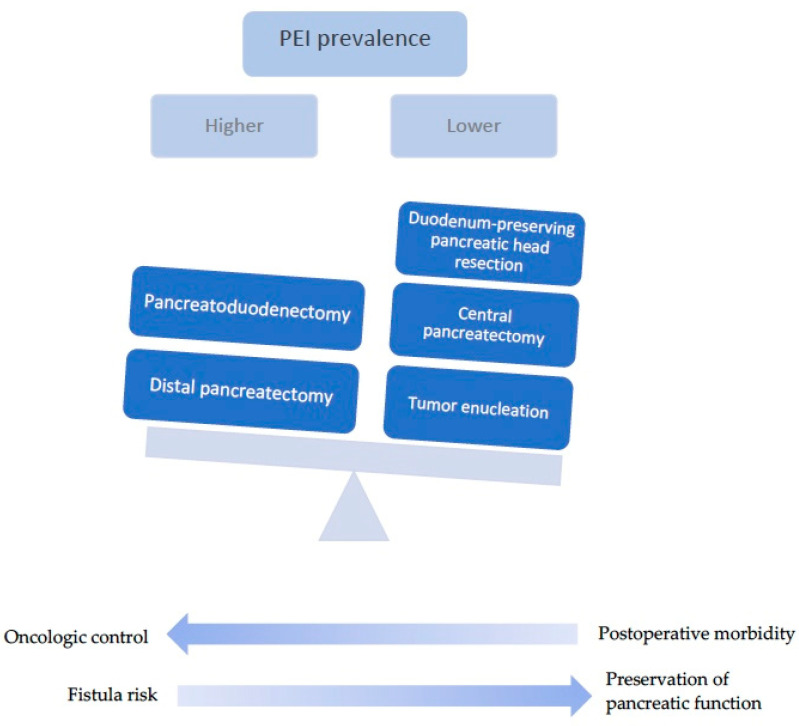
Comparison between standard pancreatic resections and parenchymal-sparing procedures, in terms of post-operative PEI rates.

The type of surgery dictates the risk of developing PEI; PEI is commonly encountered after extensive resections, while parenchymal-sparing pancreatectomies (PSP) are associated with PEI to a lower extent, as shown in Figure 3 and Table 2. Some authors have reported a threshold for the remnant pancreatic volume of 39.5% as being predictive of PEI [63]. 

Falconi et al. used the 72 h stool chymotrypsin test in order to assess pancreatic exocrine insufficiency after different types of pancreatic resections for benign lesions, revealing that PEI was more common after pancreatoduodenectomy (PD) and left pancreatectomy (LP) than after atypical pancreatic resections such as middle segment pancreatectomies (MSP) or tumor enucleations (TE) [64]. In patients who undergo PD for benign lesions, the incidence of PEI is reported to be 34–45% [65]. In contrast with standard PD, duodenum-preserving pancreatic head resections (DPPHR), owing to the spare pancreatic tissue and conservation of the duodenum and, consequently, the entero-acinar axis, have been shown to have little impact on the exocrine function, with a 6.7% prevalence of PEI in the meta-analysis by Beger et al. [62]. Of note, some surgical series have shown no difference between surgery for benign or malignant lesions [66]. Another atypical pancreatic resection is central pancreatectomy (CP), a surgical procedure that allows for the resection of benign and low-grade malignant lesions localized in the neck and proximal body of the pancreas, sparing the rest of the organ and avoiding the removal of adjacent structures; this is performed in patients that cannot benefit from tumor enucleation, or, rarely, for malignant lesions as a palliative treatment [67]. The 2013 meta-analysis by Iacono et al. showed a lower incidence of PEI after CP, at 11.9%, compared to distal pancreatectomy (DP), at 19.1%; however, higher rates of postoperative morbidity and pancreatic fistula were observed given the presence of two anastomoses [67]. More recent data also support the superiority of CP in preserving pancreatic endocrine and exocrine functions compared to DP [68,69]. This has led some authors to conclude that CP might be preferred over DP in selected cases, such as branch-duct (BD)-IPMNs, serous cystadenomas or MCNs, after a careful evaluation of pancreatic volume and a risk assessment for pancreatic fistula [70,71]. In contrast, a study comparing fecal elastase levels one year after surgery found statistically significant lower values in the CP group (151 μg/g) compared to the DP group (245 μg/g) [72].

Regarding the distal stump, the data suggest a that higher rate of PEI is associated with pancreato-jejunostomy compared to pancreato-gastrostomy (14.1% vs. 5%) [67]. 

The evident benefit of PSP regarding exocrine post-operative function comes with a greater risk of surgical complications. Thus, atypical pancreatectomies were associated with a higher incidence of pancreatic fistulas, and intra-operative and short-term morbidity [64]. Considering their advantages and disadvantages, parenchymal-sparing surgeries can be seen as providing a fine balance between increased short-term complications and the long-term conservation of exocrine and endocrine functions [73]. 

**Table 2:** Comparative overview of surgical techniques for pancreatic cystic lesions [6,74,75].

Resection Type	Details
Pancreaticoduodenectomy (Whipple’s procedure)	Resection of the pancreatic head along with the duodenum, gallbladder and distal bile duct Radical surgery with lymphadenopathy, indicated for invasive carcinoma Technically superior due to the ease of additional resection in case of positive margins in intraoperative frozen section Associated with potentially significant morbidity and high metabolic risk
Distal pancreatectomy/Left pancreatic resection	Resection of the distal portion of the pancreas Commonly associated with splenectomy Less invasive and harbors lower risk of metabolic dysfunction Carries a risk of pancreatic fistula Can limit the acquisition of further margins if the transection was done at the pancreatic neck
Duodenum-preserving pancreatic head resection	Resection of the pancreatic head with the preservation of duodenum and bile duct Indicated for benign, premalignant or low-malignant lesions of the pancreatic head Lower risk of postoperative morbidity and metabolic dysfunction Potential for pancreatic fistula
Central pancreatectomy	Segmental resection at the level of the pancreatic body Suitable for benign or low-grade malignant neoplasms High risk for pancreatic fistula
Tumor enucleation	Removal of tumor from adjacent parenchyma, with maximum preservation of pancreatic tissue Best suited for small, well delineated tumors with preoperative benign features
Total pancreatectomy	Removal of the entire pancreas Considered in diffuse disease that affects the entire parenchyma; however, even in multifocal IPMNs, only the high-risk lesion might be surgically targeted to avoid prophylactic total pancreatectomy due to profound postoperative metabolic dysfunction. Indication should also be determined based on economic and social factors (limited access to follow-up; limited insulin availability) that could additionally negatively impact metabolic outcomes.

### 4.2. The Role of the Duodenum

**Figure 4:**
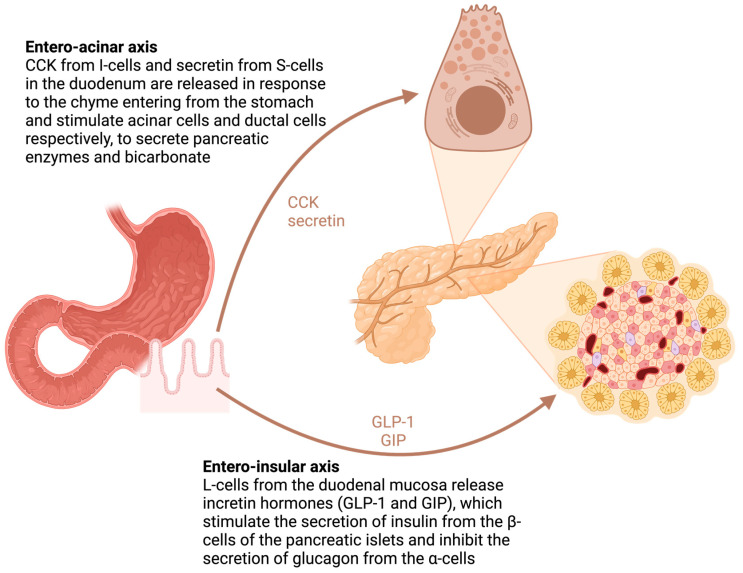
Integration of entero-acinar and entero-insular axes: duodenal signaling and pancreatic responses (Created with BioRender.com, accessed on 26 April 2024). Abbreviations: GLP-1 = glucagon-like peptide-1; GIP = glucose-dependent insulinotropic peptide; CCK = cholecystokinin.

In addition to the anatomical vicinity to the pancreas, the duodenum is a metabolically active structure with strong functional connections with the pancreas. The duodenum plays a key role as a regulator of gastrointestinal hormone secretion, such as gastrin, cholecystokinin (CCK), secretin and incretins (gastric inhibitory polypeptide (GIP) and glucagon-like peptide-1 (GLP-1)), which, in turn, are connected to the secretion of pancreatic juice and hormones, as shown in Figure 4. Surgical removal of the duodenum impairs normal secretion, leading to abnormal pancreatic exocrine and endocrine function.

In the 2022 systematic review and meta-analysis by Beger et al. [62], only 6.7% developed PEI after DPPHR, compared to 43.3% after PD (*p* < 0.01; OR: 0.15; 95%-CI: 0.07–0.32). In another paper by Beger et al., the authors show that duodenum-preserving surgery maintained responses to enterohormones compared to preoperative levels [76]. This supports the theory that duodenectomy, and not resection of the pancreatic head, is the culprit for postoperative PEI in these patients, through alterations in the enteric-mediated stimulation of pancreatic enzyme release [76]. 

A comparison between pancreatic head resection with segmental duodenectomy (PHRSD) and pylorus-preserving pancreatoduodenectomy (PPPD) for benign and low-grade malignant neoplasms of the pancreatic head revealed a higher clinically driven requirement for enzyme substitution treatment in the PPPD compared to PHRSD group [77]. 

### 4.3. PEI Evolution in Time

When considering PEI risk after pancreatic surgery, it is important to refer to the timing of PEI diagnosis. Lim et al. [78] demonstrated that the 30-day outcome reporting of PEI is inadequate, as the risk can be significantly underestimated—there is an increasing rate of PEI with continuing follow-up of patients: 21% within 30 days after surgery, 31% between 30 and 90 days and 48% after 90 days [78]. Similarly, a retrospective study by Kusakabe et al. including patients who underwent PD or DP found a mean time to PEI onset at 14.2 ± 26.9 (IQR: 0.89–12.69) months [66]. Among the risk factors for developing PEI were race, lower BMI, family history of diabetes mellitus (DM), elevated pre-operative bilirubin and PD [66].

### 4.4. The Impact of PEI after Surgery

It is well-recognized that PEI has an important negative impact on the daily life of patients, frequently leading to social stigma. Shah KP et al. [13] analyzed patient-reported outcomes after pancreatic resection for cystic neoplasms, revealing that 55% of patients had steatorrhea, 41% had floating stools, 14% had oily/greasy stools or oil drops in the toilet and 25% presented abnormal stool color. Bloating after meals was noted in 27% of patients, with another 10% reporting cramping after meals. However, only 7.8% of patients were taking pancreatic enzymes [13]. Another study, by Fong et al., analyzed the quality of life after PD in 245 patients, of whom 157 (64.1%) were operated for nonmalignant lesions, and revealed that 50.4% of responders were taking pancreatic enzyme replacement therapy [79]. 

Despite being a frequent complication of pancreatic surgery, PEI is under-recognized and under-treated, exposing patients to nutritional risks, which can impact survival, tolerance and fitness for further oncologic treatments [80,81,82]. Clinical suspicion or evidence of PEI should prompt pancreatic enzyme supplementation after pancreatic resection. The follow-up of PEI in resected patients is guided by clinical and nutritional parameters [81].

## 5. Metabolic Dysfunction—Diabetes and Hepatosteatosis

Metabolic dysfunction, comprising steatotic liver disease (SLD) and DM, is another significant long-term morbidity after pancreatic surgery, which is dependent on the type of resection that is performed. Considering the interventions with the most severe metabolic impact, NOD can be seen in up to one-third of patients undergoing pancreatectomy for a pancreatic cystic neoplasm and SLD in about one in four patients [13,62]. Similar to the mechanism of PEI in pancreatic head resections, duodenal resection plays a major role in endocrine insufficiency after PD [76]. Some have theorized that there are different metabolic consequences according to resection type depending on islet density in the resected segment, considering the higher density of β-cells in the tail region [83,84,85]. There are also other mechanisms to consider, such as islet cell plasticity and the trans-differentiation of exocrine ductal and acinar cells, which can contribute to maintaining the β-cell mass [86]. On the other hand, there might be a two-way link between pancreatic cystic lesions and DM, similar to that seen in PDAC—the resection of a pre-malignant or malignant PCL might induce an improvement in or resolution of a paraneoplastic-induced DM [87]. 

As with PEI, metabolic dysfunction after pancreatic surgery can impact the nutritional status of patients, as well as the risk for postoperative complications and even the survival rate [88]. 

### 5.1. Diabetes after Pancreatic Resection

Type 3 DM or pancreatogenic diabetes is known to occur in the setting of pancreatic pathology, including resections [89]. Glycemic metabolic disturbances were reported in various grades after standard and parenchymal-sparing pancreatic resections for benign lesions. Scholten et al. found an overall prevalence of NOD after PD of 16%, with 6% developing insulin-dependent NOD [90]. In the 2020 systematic review and meta-analysis conducted by Beger et al. [76], pooled data from 386 patients undergoing either PD or DPPHR showed an incidence of NOD of 15% vs. 6%, respectively (*p* = 0.007; OR 3.01; 95%CI: 1.39–6.49), after 52.2 ± 33.5 months follow-up. The metabolic benefit of DPPHR was doubled by the benefits obtained in exocrine function preservation, with PEI seen in only 6.8% versus 44.9% at 20.1 ± 22.2 months follow-up (*p* < 0.001; OR 7.03; 95% CI: 3.20–15.41). By measuring the fasting and stimulated hormone levels after surgery, a significant reduction in insulin, pancreatic polypeptide (PP), GIP and CCK secretion was seen in PD compared to DPPHR, most likely caused by the loss of duodenal entero-endocrine cells. Although pancreatic head resection reduces the functional parenchyma by approximately 40%, it causes only a modest reduction in the endocrine functions compared to duodenectomy, again highlighting the crucial role of the duodenum as a key metabolic and signaling organ. The significant increase in GLP-1 and glucagon levels in PPPD may be caused by hyper-functional or transdifferentiated endocrine cells within the lower small intestine, as extrapancreatic sites of glucagon synthesis are already documented in humans [91].

Interestingly, PPPD was associated with significantly higher basal and meal-stimulated gastrin, secretin and CCK levels, but had no impact on NOD incidence. Evidence showed that the distension of the pyloric antrum may be a potent stimulus of gastrin release, with further implications for pancreatic enzyme release [92]. Also, the interruption of gastric antral and pancreatic neural connections may lead to a loss of vagus-sensitive humoral factors, as the pyloric canal possesses complex distributions and specializations of vagal endings, with mechanoreceptors having the potential to generate gut reflexes and gastrointestinal hormone release [93]. The 2022 updated systematic review and meta-analysis by Beger et al. [62] of pooled data from 2729 patients with a mean follow-up of 32 months, looking at the prevalence of pancreatic exocrine and endocrine dysfunction and hepatic steatosis after different types of pancreatic resections, showed that NOD was diagnosed in 15.7% following PD vs. 5% following DPPHR; a similar incidence of NOD was seen after PPPD and TE—19.7% and 5.7%, respectively. Patients with left pancreatic resection (LP) had a significantly higher incidence of NOD than those with pancreatic middle segment resection (PMSR)—23.3% vs. 5.6% (*p* < 0.01; OR: 0.20; 95%-CI: 0.12–0.32). After PD/PPPD, 23.8% developed non-alcoholic fatty liver disease (NAFLD), compared to 3% after DPPHR (*p* < 0.01), at a mean follow-up of 30.4 months. 

### 5.2. Steatotic Liver Disease

De novo SLD after pancreatectomy has distinct clinical features, including the absence of traditional risk factors like metabolic syndrome or obesity, and the presence of malnutrition and malabsorption as a result of PEI. Therefore, the extension of pancreatic resection was found to be an independent risk factor for the development of SLD, being correlated with PEI due to loss of pancreatic tissue [94]. The pathophysiology and exact molecular mechanisms leading to post-pancreatectomy SLD are poorly understood. Nevertheless, in contrast to traditional NAFLD, individuals experiencing de novo NAFLD following pancreatectomy exhibit malnutrition, characterized by a lower BMI, decreased levels of serum cholesterol and albumin and an enhanced response to PERT [95]. Several experimental and human studies have demonstrated that changes in lipid metabolism caused by deficiencies in lipoatrophic agents and the enhanced expression of lipogenesis genes could contribute to the pathogenesis of SLD [96]. Hypocarnitinemia was documented in 61.9% of patients undergoing pancreatectomy, with a subsequent high ratio of acyl to free carnitine [97]; this finding was associated with hepatic steatosis as a result of mitochondrial dysfunction, leading to decreased fatty acid oxidation and impaired lipid metabolism. 

A randomized multicenter clinical trial showed that patients receiving high-dose PERT starting one week after subtotal stomach-preserving PD had a significantly lower incidence of de novo NAFLD at 12 months follow-up (*p* < 0.001). The same group exhibited significantly higher serum concentrations of total protein, albumin, pre-albumin, cholinesterase and total cholesterol [98]. 

A recent systematic review described an overall maximum incidence rate of 66% de novo NAFLD diagnosed within 12 months of pancreatectomy for mixed (both benign and malignant) pathology. Regarding surgical technique, the pooled data showed an incidence of de novo NAFLD after PD for benign lesions of 16–26% [99]. In addition to the resection type, other risk factors for NAFLD occurrence were residual pancreatic volume, pancreatic exocrine and endocrine dysfunction and post-operative nutritional management [99]. Patel V. et al. also investigated the incidence, time to diagnosis and perioperative risk factors of de novo NAFLD in a single-center retrospective cohort study including patients who underwent pancreatectomy for both benign and malignant pathologies between 2000 and 2020 [100]. The overall incidence of de novo NAFLD was 17.5%, with a two-fold higher incidence in patients with malignant compared to benign pancreatic disease (21.3% vs. 9.5%) and a significantly shorter (by an average of 6 months) time to diagnosis compared to the benign group (26.4 vs. 32.8 months, *p* = 0.03). When looking at the surgical technique for benign pancreatic lesions, PD was associated with a higher incidence of de novo NAFLD compared to DP (11.1% vs. 8.3%), with a non-significant difference in average time to diagnosis (33.9 PD vs. 31.9 months). After multivariate analyses, pre-operative BMI was an independent risk factor for de novo NAFLD (*p* = 0.03), regardless of the surgical indication, type of surgery or other metabolic risk factors. The overall incidence of NOD in patients with benign lesions was 42.9%, with a higher incidence after DP compared to PD (54.1%, vs. 27.8%). Additionally, postoperative BMI was significantly lower in patients undergoing PD vs. DP for benign disease (*p* = 0.02), again highlighting the implications of duodenal resection in the development of malnutrition and metabolic disturbances.

Li et al. analyzed the impact of partial pancreatectomy on the incidence of NAFLD in patients with IPMN using MRI-enabled liver fat signal fraction (LFSF) tracking. Out of 49 patients, 34% developed SLD post-surgery. The entire cohort experienced notable weight loss (*p* < 0.01). Following surgery, a substantial rise in LFSF was observed: 1.3% vs. 9.6% after PD (*p* < 0.01) and 2.1% vs. 9.4% after DP (*p* = 0.01). [101] 

Another study, looking at pancreatectomies for pancreatic cystic lesions only, revealed an NOD prevalence of 9.1%, 15.1% and 20.2% at 6, 12 and 24 months, respectively, after resection, with no differences in LP vs. PD. On multivariate analysis, predictors for NOD with corresponding adjusted hazard ratios were advanced age (1.97), obesity (2.63), hypertension (1.79) and cardiovascular disease (2.54) [102].

Summarizing the data on exocrine and endocrine pancreatic dysfunction after surgery, PD and LP were associated with the highest incidence of NOD and PEI compared to duodenum-preserving techniques, PMR and TE, as shown in Table 3. Duodenum-sparing resection was also associated with a lower incidence of NAFLD compared to PD. Growing evidence suggests that duodenectomy rather than pancreatic head resection is associated with long-term metabolic disturbances and PEI after pancreatic surgery for benign lesions, with a possible explanation for this being the pivotal role of the duodenum in the intestinal nutrient sensing and the release of hormones with pancreatic trophic and metabolic activity [65,76]. De novo post-pancreatectomy NAFLD may be a result of malabsorption/malnutrition secondary to PEI, and early high-dose PERT to improve nutritional status may decrease the incidence of hepatic steatosis after pancreatic resection and may positively impact the survival rate, although future clinical studies are needed for confirmation, as well as for determining the optimal dose and duration of PERT to prevent these metabolic dysfunctions. Additionally, high pre-operative BMI and glycated hemoglobin (HbA1c), older age and cardiovascular comorbidities seem to be significant predictors for metabolic complications after surgery, highlighting a group that needs intensive counselling and close surveillance after pancreatic resection [89,102].

Studies evaluating the quality of life after pancreatic surgery for benign lesions are scarce, with most of them evaluating patient outcomes after pancreatectomy for PDAC. A study analyzing changes in quality of life after different types of pancreatic resections concluded that although TP and PD had comparable impacts on quality of life, patients experienced an extended duration before returning to their preoperative or baseline status following TP. Quality of life was enhanced post DPPHR compared to PD. Nevertheless, the debate persists regarding the quality of life among individuals who underwent CP versus PD. The primary factor influencing QOL was the decline in exocrine and endocrine functions post-surgery, and minimally invasive procedures demonstrated potential in enhancing patients’ quality of life during the initial phases after PD and DP [103]. Gastrointestinal manifestations such as bloating and indigestion play a major role in impacting long-term quality of life. Some of these manifestations can be attributed to PEI following PD rather than complications arising post-surgery, and PEI further increases the incidence of metabolic dysfunction, thus playing a pivotal role in short-term and long-term outcomes, including quality of life and survival [104]. Research has additionally demonstrated that there is a correlation between preoperative reduction in body weight, compromised preoperative pancreatic exocrine function and an extended duration of the surgical procedure and delays in quality of life improvements [105]. 

## 6. Limitations

There are several limitations with regard to the studies reporting on postoperative surveillance of PCLs. Along with the retrospective nature of surgical series, there is significant heterogeneity in the definition of recurrence and the follow-up protocol. Also, the use of biomarkers in the surveillance of resected cysts was reported in only a few studies. Notably, data about PEI and metabolic dysfunction after pancreatic surgery are mostly from series including resections for a wide range of lesions, and not PCLs specifically. Future research should also address surveillance after emerging therapeutic options such as EUS-guided injection or ablation therapies.

## 7. Conclusions

The surveillance of resected PCLs is required because of the recurrence risk in the remnant pancreas, as well as systemic progression and the long-term morbidity represented by exocrine and endocrine insufficiency. Although several factors, both patient- and surgery-related, can refine these risks, there is a need to better define features that allow for a more precise risk-based surveillance of resected pancreatic cysts.

## Figures and Tables

**Table 1:** Recommendations of the International Evidence-based Kyoto Guidelines for the surgical management of IPMNs.

	Type of Surgery	Partial Pancreatectomy	Radical Pancreatectomy with Lymph Node Dissection	Organ-Preserving Pancreatectomy without Lymphadenectomy *	Comments
IPMN Subtype					
BD-IPMN		Usually preferred	Only when IC is suspected or confirmed	Only if the suspicion for IC is low based on preoperative features and/or intraoperative findings	Minimally invasive approaches (laparoscopic or robotic pancreatectomy) can be utilized. Goal: negative surgical margins ** Pancreatoscopy should be performed preoperatively, but is not recommended as a routine examination.
Mixed IPMN			Same as for BD-IPMN	Same as for BD-IPMN	
MD-IPMN			Same as for BD-IPMN	Same as for BD-IPMN	

Abbreviations: IC = invasive carcinoma, BD-IPMN = branch duct-IPMN; MD-IPMN = main duct-IPMN; * Middle pancreatectomy (MP) or spleen-preserving distal pancreatectomy (DP). ** Intraoperative frozen section recommended to rule out unexpected MPD involvement or neoplasia at resection margin (irrespective of complete macroscopic resection): if IC or HGD are present, additional resection is recommended; if normal epithelium or low-grade dysplasia (LGD), additional resection is not necessary. The absence of epithelial cells at the transection margin is not equivalent to a negative margin, and additional resection should be considered. Leaving HGD at the margin may be appropriate to avoid a total pancreatectomy, particularly in older or frail patients, as the prognosis is dictated by IC.

**Table 3:** Incidence of SLD/NAFLD, NOD and PEI according to different types of pancreatic resections.

Surgery Type	NOD (%)	Steatosis/NAFLD (%)	PEI (%)	Other Postop. Findings
PD	15 (*Beger, 2020* [76]) 15.7 (*Beger, 2022* [62]) 27.8 (*Patel, 2023* [100]) 9–24 (*Wu, 2020* [89])	23.8 (*Beger, 2022*) 16–26 (*Shah P, 2022*) 11.1 (*Patel, 2023*)	44.9 (*Beger, 2020*) 44.3 (*Beger, 2022*) 44.4 (*Patel, 2023*)	Significant decrease in fasting basal and stimulated levels of gastrin, motilin, insulin, C-peptide, secretin, PP and GIP after mean 7.8 mo. Significantly lower levels of gastrin, secretin and CCK compared to PPPD (*p* < 0.05) Stimulated CCK secretion is significantly reduced compared to PPPD (*p* < 0.0001) and DPPHR (*p* = 0.011) (*Beger, 2020*)
DPPHR	6 (*Beger, 2020*) 5 (*Beger, 2022*)	3 (*Beger, 2022*)	6.8 (*Beger, 2020*) 6.7 (*Beger, 2022*)	Normal levels of fasting motilin and secretin; stimulated response of insulin, gastrin, motilin, CCK and secretin comparable to preop. (*Beger, 2020*)
LP/DP	23.3 (*Beger, 2022*) 54.1 (*Patel, 2023*) 3–40 (*Wu, 2020*)	8.3 (*Patel, 2023*)	17 (*Beger, 2022*) 25 (*Patel, 2023*)	
PPPD	19.7 (*Beger, 2022*)			Significantly increased fasting basal and stimulated secretion of GLP-1 and glucagon (*p* < 0.05) (*Beger, 2020*)
PMSR/CP	5.6 (*Beger, 2022*) 0–14 (*Wu, 2020*)		8 (*Beger, 2022*)	

Abbreviations: SLD = steatotic liver disease; NAFLD = non-alcoholic fatty liver disease; NOD = new-onset diabetes; PEI—pancreatic exocrine insufficiency; PD = pancreatoduodenectomy; DPPHR = duodenum-preserving pancreatic head resections; PPPD = pylorus-preserving pancreatoduodenectomy; PMSR = pancreatic middle segment resection; LP = left pancreatectomy; DP = distal pancreatectomy; CP = central pancreatectomy.

## Data Availability

Not applicable.
